# Challenging features of left ventricular wall thickening in a young patient with multiple myeloma and shock: when magnetic resonance imaging makes the difference—a case report

**DOI:** 10.1093/ehjcr/ytaf590

**Published:** 2025-11-25

**Authors:** Carmine Galdieri, Stefania Sacchi, Magda Marcatti, Silvia Ajello, Anna Mara Scandroglio

**Affiliations:** Cardiac Intensive Care Unit, San Raffaele University Hospital, Via Olgettina 60, Milan 20132, Italy; Cardiac Intensive Care Unit, San Raffaele University Hospital, Via Olgettina 60, Milan 20132, Italy; Department of Hematology, San Raffaele University Hospital, Via Olgettina 60, Milan 20132, Italy; Cardiac Intensive Care Unit, San Raffaele University Hospital, Via Olgettina 60, Milan 20132, Italy; Cardiac Intensive Care Unit, San Raffaele University Hospital, Via Olgettina 60, Milan 20132, Italy

**Keywords:** Left ventricular wall thickening, Multiple myeloma, Cardiogenic shock, Cardiac MRI, Case report

## Abstract

**Background:**

Left ventricular walls may thicken for various reasons in patients with multiple myeloma, and identifying the cause is crucial for targeted treatment, especially in the context of cardiogenic shock. While echocardiography may not provide a definitive diagnosis, cardiac magnetic resonance imaging (MRI), which allows for specific characterization of structural changes in the myocardium, can be diagnostic.

**Case summary:**

A 44-year-old Caucasian man with multiple myeloma, recently undergone autologous stem cell transplantation with following medullary aplasia, developed cardiogenic shock. The identification of the underlying cause was challenging since echocardiographic imaging, showing severe thickening of left ventricle walls, was not able to discriminate between coexisting amyloid light-chain (AL) amyloidosis and myocarditis features. Conversely, cardiac MRI imaging played a major role in diagnosing myocarditis. Endomyocardial biopsy was not performed due to severe thrombocytopenia and increased bleeding risk. After initial circulatory support and empiric treatment, the patient ultimately experienced progressive myocardial recovery.

**Discussion:**

In patients with multiple myeloma and shock, acute myocarditis and AL cardiac amyloidosis can show similar echocardiographic features yet demand different treatments and lead to varying outcomes. Cardiac MRI plays a crucial role in the diagnostic workup, offering detailed insights that guide targeted management.

Learning points

- Left ventricular wall thickening in cardiogenic shock patients with multiple myeloma and recent stem cell transplantation is a challenging finding.
- Echocardiography alone may not differentiate between hypertrophy, amyloid light-chain amyloidosis, or myocarditis, conditions with distinct treatments and prognoses, while cardiac magnetic resonance imaging may be diagnostic, helping guide appropriate therapeutic decisions.

## Introduction

Multiple myeloma can present with a range of symptoms, from asymptomatic cases to severe organ dysfunction, with risks including cardiac structural changes, such as hypertrophy or amyloidosis, and infection, especially after autologous stem cell transplantation.^1–4^ In this complex context, especially in the presence of left ventricular (LV) wall thickening, identifying the cause of haemodynamic deterioration is crucial. While echocardiography remains a valuable initial tool, it may be limited in providing a definitive diagnosis in such scenarios.^5,6^ Cardiac magnetic resonance imaging (MRI), with its advanced myocardial tissue characterization capabilities, offers a significant advantage by identifying distinct patterns that help differentiate between conditions such as cardiac amyloidosis and myocarditis, both of which may be associated with multiple myeloma.^5,6^ These conditions have unique prognostic implications and require tailored therapeutic strategies. By revealing specific myocardial substrate patterns, such as diffuse subendocardial or transmural involvement in amyloid light-chain (AL) amyloidosis, or subepicardial and mid-wall oedema in myocarditis, cardiac MRI can provide a definitive diagnosis and play a pivotal role in guiding the optimal treatment approach for the patient.^7^ This case report highlights the diagnostic utility of cardiac MRI in a multiple myeloma patient presenting with cardiogenic shock following autologous stem cell transplantation.

## Summary figure

**Figure:**
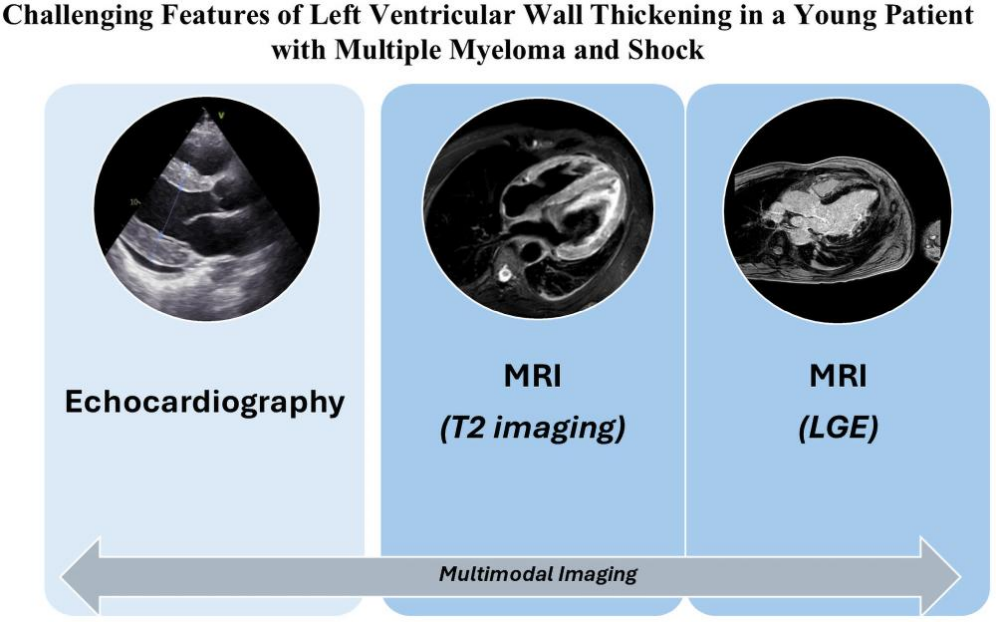


## Case presentation

A 44-year-old man with multiple myeloma, post-autologous stem cell transplantation, was admitted to the Cardiac Intensive Care Unit with haemodynamic deterioration, dyspnoea, and oliguria. His past medical history was unremarkable for cardiac disease.

## Diagnostic findings

**Figure 1:**
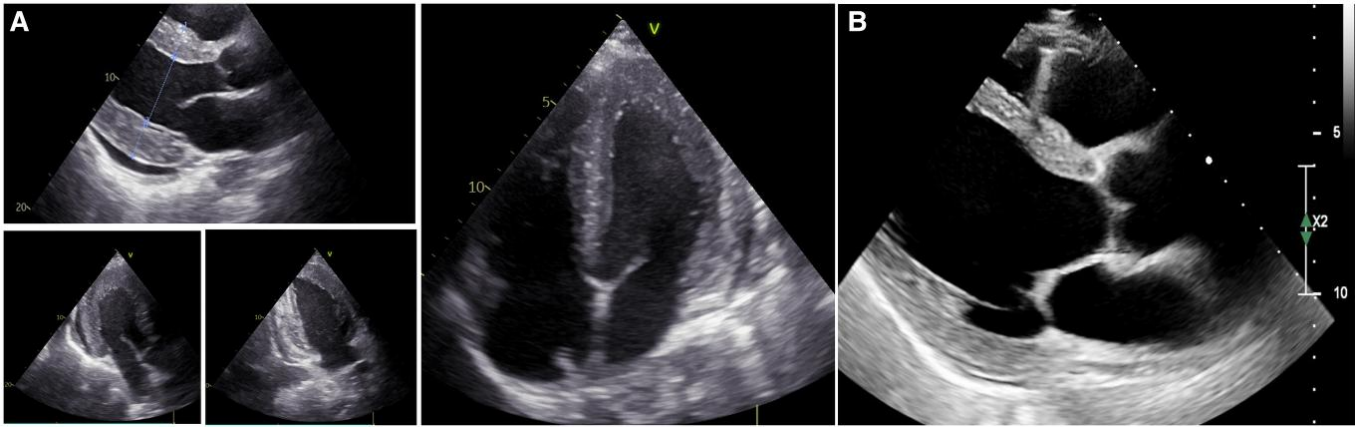
Echocardiography. (*A*) Upon arrival, transthoracic echocardiogram revealed severe concentric left ventricular hypertrophy, with an IVS wall thickness of 22 mm and a left ventricular posterior wall thickness of 20 mm, along with severely decreased biventricular function and a mild pericardial effusion. (*B*) Follow-up transthoracic echocardiogram showed a reduction in left ventricular thickness (IVS of 13 mm) and a mild improvement in left ventricular ejection fraction.

On admission, electrocardiogram showed atrial fibrillation with ST segment and T wave abnormalities. Transthoracic echocardiogram (TTE) revealed significant LV hypertrophy (IVS-interventricular septum 22 mm; PW-posterior wall 20 mm), severe biventricular dysfunction, and mild pericardial effusion, raising suspicion for both infiltrative and inflammatory processes (*Figure 1A*).

Blood tests showed elevated troponin T (5000 pg/mL), N-terminal pro–B-type natriuretic peptide (20 000 ng/L), and lactate (11 mmol/L), with mild C-reactive protein elevation. Additionally, the patient exhibited severe lymphocytopenia (0.6 × 10^9/L), thrombocytopenia (30 × 10^9/L), and evidence of hepato-renal organ dysfunction. Haemodynamic monitoring indicated severe cardiac dysfunction [cardiac index (CI), 1.3 L/min/m²; pulmonary capillary wedge pressure (PCWP), 20 mmHg; cardiac power output (CPO), 0.7]. Blood cultures were negative. Serological tests revealed positive IgM and IgG for cytomegalovirus (CMV), with elevated CMV DNA copies (14 400 U/mL) (*Table 1*).

**Table 1.** 

Variable	Reference range for adults	On arrival at ICU
Laboratory data^a^		
Haemoglobin (g/dL)	12.0–16.0	8.7
Haematocrit (%)	36.0–46.0	26.1
White cell count (per μL)	4500–11 000	19 600
Differential count (%)		
Neutrophils	40–70	74.2
Lymphocytes	22–44	2.9
Monocytes	4–11	19.2
Eosinophils	0–8	3.5
Platelet count (per μL)	150 000–400 000	30 000
Sodium (mmol/L)	135–148	151
Potassium (mmol/L)	3.5–5	4.6
Urea (mg/dL)	10–50	73
Creatinine (mg/dL)	0.5–1.2	2.3
Glucose (mg/dL)	60–100	170
Lactate (mmol/L)	0.5–2.0	11
Ionized calcium (mmol/L)	2.1–2.6	2.5
Total bilirubin (mg/dL)	0.1–1.0	2.2
Direct bilirubin (mg/dL)	0.01–0.25	1.38
Alanine aminotransferase (IU/L)	6–59	2402
Aspartate aminotransferase (IU/L)	5–35	3475
Albumin (g/L)	35–50	32
Creatine kinase (IU/L)	20–195	509
*n*-Terminal pro-B-type natriuretic peptide (pg/mL)	0–88	20 000
High-sensitivity troponin T (ng/L)	0–14	5000
Lactate dehydrogenase (IU/L)	125–220	226
C-reactive protein (mg/L)	<6	50
Thyroid-stimulating hormone (μIU/mL)	0.25–5	1.21
Anti-herpes simplex virus 1 (HSV-1) antibodies IgG	Negative	Positive
Anti-herpes simplex virus 1 (HSV-1) antibodies IgM	Negative	Negative
Adenovirus DNA (copies/mL)	Negative	Negative
HCV RNA (qualitative) (UI/mL)	<15	<15 UI/mL
Anti-HIV-1 antibodies (UI/mL)	Negative	Negative
HBsAg (Australia Antigene)	Negative	Negative
Anti-cytomegalovirus (CMV) antibodies IgM (U/mL)	Negative	277
Anti-cytomegalovirus (CMV) antibodies IgG (U/mL)	Negative	0.19
Cytomegalovirus DNA (copies/mL)	Negative	14 440
Right heart catheterization^b^		
Cardiac index (L/min/m^2^)	2.5–4.0	1.3
Cardiac power output		0.7
Pulmonary artery pulsatility index (PAPi)		0.6
Mixed venous saturation (%)	60–80	55
Right atrial pressure (mmHg)	0–5	20
Mean pulmonary artery pressure (MPAP) (mmHg)	6–12	30
Pulmonary artery wedge pressure (PAWP) (mmHg)	10–20	18
Systemic vascular resistance (SVR) (WU)	10–15	23
Pulmonary vascular resistance (PVR) (WU)	<3.1	2.0

^a^Reference values are affected by many variables, including the patient population and the laboratory methods used. The ranges used at our hospital for adults who are not pregnant and do not have medical conditions that could affect the results. They may therefore not be appropriate for all patients.

^b^Normal range values of haemodynamic parameters for adults are taken from ‘Quick Guide to Cardiopulmonary Care’ Edwards.

**Figure 2:**
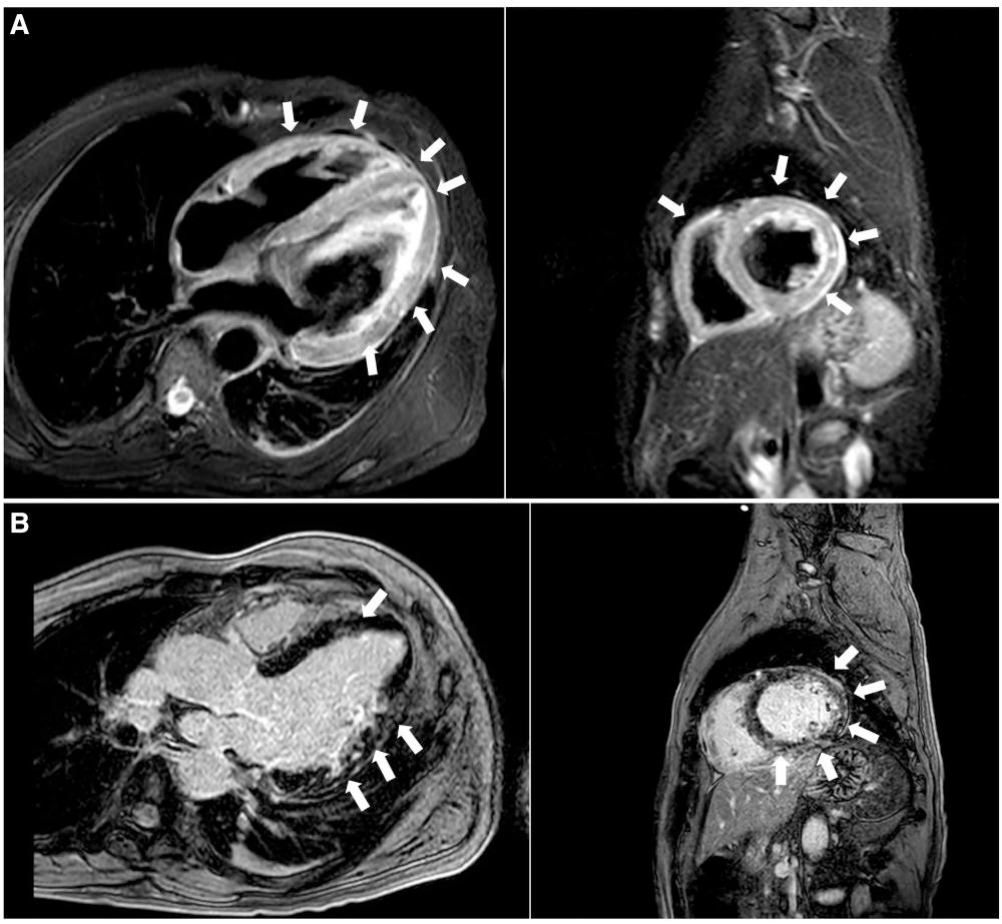
Cardiac magnetic resonance imaging. (*A*) Long- and short-axis T2-weighted short tau inversion recovery images showed diffuse myocardial hyperintensity (marked with arrows) consistent with widespread myocardial oedema, with more intense patchy areas in the basal-to-mid-lateral wall (mean T2 relaxation times, 69 ms; normal ≤ 50 ms) (*Figure 2A*). Additional tissue characterization parameters were consistent with widespread myocardial tissue injury (mean T1 relaxation times, 1172 ms; normal < 1045 ms; mean elevated extracellular volume fraction, 44%; normal < 27%). (*B*) In the long- and short-axis late gadolinium enhancement sequences, multiple striae and patchy areas of late gadolinium enhancement with non-ischaemic distribution (mid-wall, subepicardial, and septal) were identified, particularly in the basal-to-mid-lateral wall. Late gadolinium enhancement, highlighted by arrows, is evident in the basal to mid-lateral and inferolateral segments of the left ventricular myocardium (*Figure 2B*).

Cardiac MRI confirmed severe biventricular dysfunction [left ventricular ejection fraction (LVEF), 35%; right ventricular ejection fraction (RVEF), 39%], diffuse myocardial oedema, elevated extracellular volume (ECV) due to necrosis, and interstitial fibrosis, along with concurrent microvascular damage (*Figure 2A*). Late gadolinium enhancement (LGE) was observed in the basal to mid-lateral and inferolateral wall segments of the left ventricle (*Figure 2B*), findings consistent with viral myocarditis, effectively ruling out cardiac amyloidosis as the cause of hypertrophy.

## Management and outcome

The patient received circulatory support, including high-dose adrenaline and norepinephrine, along with the insertion of a bedside intra-aortic balloon pump, as well as ganciclovir and broad-spectrum antibiotics.

Over the course of several days, he showed improvement in haemodynamics, reduction in ventricular wall thickness likely from resolving inflammation (*Figure 1B*), and decreased CMV DNA levels. Given the patient’s high bleeding risk, the diagnostic role of cardiac MRI, and the favourable response to therapy with progressive resolution of LV hypertrophy and biventricular dysfunction, the patient was not subjected to an endomyocardial biopsy.

Finally, the patient was discharged from the hospital with full recovery of cardiac function.

## Discussion

Multiple myeloma is a heterogeneous disease with clinical presentations that range from an indolent course to rapidly progressive symptoms and organ dysfunction.^1^ In the present case, the patient’s clinical presentation was marked by severe cardiac dysfunction leading to cardiogenic shock.

Patients with multiple myeloma are at risk of cardiac structural changes characterized by thickening of the ventricular walls, since AL amyloidosis with cardiac involvement can coexist.^2,3^ When cardiac AL amyloidosis presents with cardiogenic shock, mortality rate is high, despite aggressive treatment with inotropic and mechanical circulatory support.^2,3^

Patients with multiple myeloma are also at a high risk of infection due to multifactorial immunodeficiency caused by the disease itself and treatment regimens during various phases of therapy, particularly following autologous haematopoietic cell transplantation and engraftment.^1,4^ Among these, CMV infection is common^4^ and peri-myocarditis has been previously described.^8^

The clinical manifestations of myocarditis can vary widely. In some cases, it may present as subclinical disease, while in others, it leads to acute fulminant myocarditis and cardiogenic shock.^7,8^ Cardiogenic shock may occur around 2 weeks after a distinct viral prodrome.^7,8^ Patients have severe cardiovascular compromise and may require circulatory support.^8^ Multiple foci of active lymphocytic myocarditis are common, and ventricular systolic dysfunction often normalized in patients surviving the acute illness.^8^ All these patterns were consistently observed in our patient with multiple myeloma and CMV infection, who was ultimately diagnosed with cardiac dysfunction, leading to cardiogenic shock caused by fulminant myocarditis.

The final diagnosis was challenging because the echocardiographic features on admission, including significantly thickened ventricular walls and severe global ventricular dysfunction, did not aid in distinguishing between fulminant myocarditis and AL cardiac amyloidosis.

Although the hypothesis of AL cardiac amyloidosis was less probable given the patient’s silent cardiac history and previous normal echo findings, MRI was the diagnostic technique that enabled the detection of myocarditis features.^8^

The expansion of extracellular volume due to amyloid fibril deposition is effectively visualized using gadolinium-based contrast on cardiac MRI. Late gadolinium enhancement in amyloidosis shows a characteristic, diffuse subendocardial and/or transmural pattern, often coupled with ‘abnormal gadolinium kinetics’ where gadolinium and the blood null simultaneously.^6^

In contrast, myocarditis is typically characterized by myocardial oedema, visible on T2-weighted imaging, along with hyperaemia, increased vascular permeability, and interstitial expansion, leading to prolonged T1 relaxation time and increased gadolinium uptake. Myocyte injury from inflammation causes necrosis, fibrosis, and scarring, with LGE often seen in the subepicardial or mid-myocardial layers, predominantly in the basal to mid-lateral and inferolateral segments of the left ventricle.^5^

In this case, the CMR findings were consistent with acute myocarditis, and these findings were crucial in guiding the clinical management, effectively ruling out amyloidosis.

In clinical practice, endomyocardial biopsy is often vital for diagnosing myocarditis.^8^ It is recommended especially in the presence of haemodynamic compromise with persistent instability, incessant or life-threatening arrhythmias, or when conventional diagnostic methods (e.g. MRI) do not provide a definitive diagnosis. The procedure can also help in distinguishing between viral, autoimmune, or other causes, aiding in tailored treatment decisions.^8^ However, when the procedure carries a high risk due to the patient’s condition, in addition to the inherent risks of the procedure itself, and a specific aetiology is strongly suspected with a favourable clinical response to treatment, the use of biopsy is debated.^8^ In this case, the high procedural risk, compounded by the patient’s elevated bleeding risk due to coagulopathy, along with the presence of CMV DNA, diagnostic CMR findings, and a positive therapeutic response, supported the diagnosis, eliminating the need for biopsy.

In cases of acute myocarditis with haemodynamic compromise, management involves a multifaceted approach.^8,9^ Haemodynamic stabilization is achieved using inotropic agents or mechanical circulatory support, such as intra-aortic balloon pumps or LV assist devices.^9^ Addressing the underlying aetiology is crucial, with antiviral therapy for viral myocarditis or immunosuppressive treatment for autoimmune forms.^8^ In autoimmune myocarditis, corticosteroids and other immunosuppressive agents are considered, though their use remains controversial and should be reserved for cases with clear indications.^8,9^ In this case, the acute viral infection prompted the administration of ganciclovir. Ganciclovir is a well-established antiviral treatment in managing severe CMV infections in immunocompromised patients, and its use in this case, alongside the circulatory support, was effective in managing the myocarditis and facilitating quick myocardial recovery.^7–9^

## Conclusions

This case highlights how patients with multiple myeloma can present with severe ventricular dysfunction and cardiogenic shock. Acute myocarditis and AL amyloidosis can present with similar echocardiographic features but require different treatments and have distinct outcomes. A thorough diagnostic workup, including multimodal imaging, is essential. Cardiac MRI plays a pivotal role in such cases, providing detailed myocardial tissue characterization to guide accurate diagnosis and appropriate management.

## Lead author biography


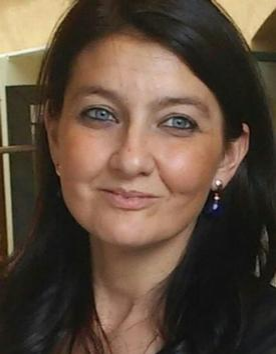


Dr Stefania Sacchi graduated in Medicine and Surgery and obtained her Ph.D. in Clinical Sciences, specializing in heart failure. She completed a research fellowship abroad at Imperial College London. She is a clinical cardiologist with expertise in coronary intensive care, heart failure, and arrhythmology and currently works as a consultant cardiologist in the Coronary Care Unit at San Raffaele Hospital in Milan, Italy.

## Data Availability

The data underlying this article are available in the article.
